# Supplementary material for: Atraric Acid Ameliorates Hyperpigmentation through the Downregulation of the PKA/CREB/MITF Signaling Pathway
Source: Int J Mol Sci. 2022 Dec 15;23(24):15952. doi: 10.3390/ijms232415952 (PMC9788525; doi:10.3390/ijms232415952)
Supplement: Supplementary file 1 [file ijms-23-15952-s001.zip › ijms-1957596-supplementary.pdf]

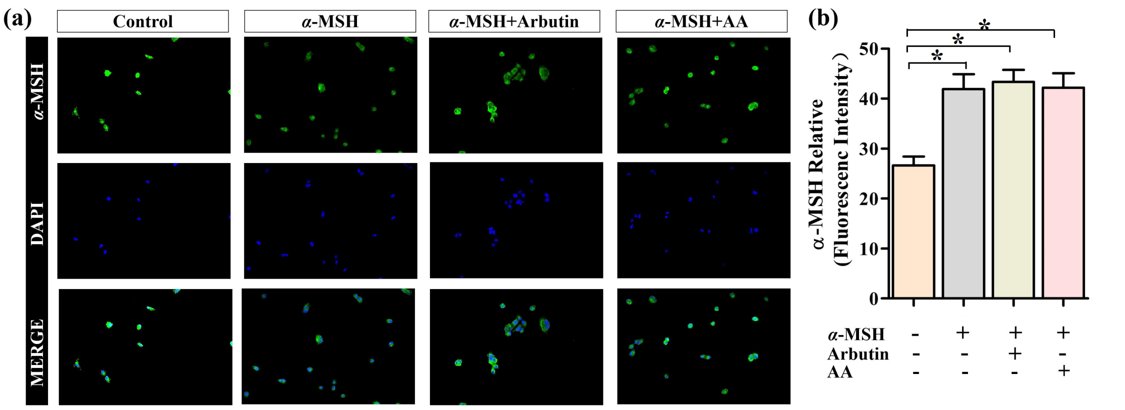

Figure S1 The images and optical density of α-MSH protein (a). The α-MSH relative fluorescence intensity (b). (n = 3 per group).

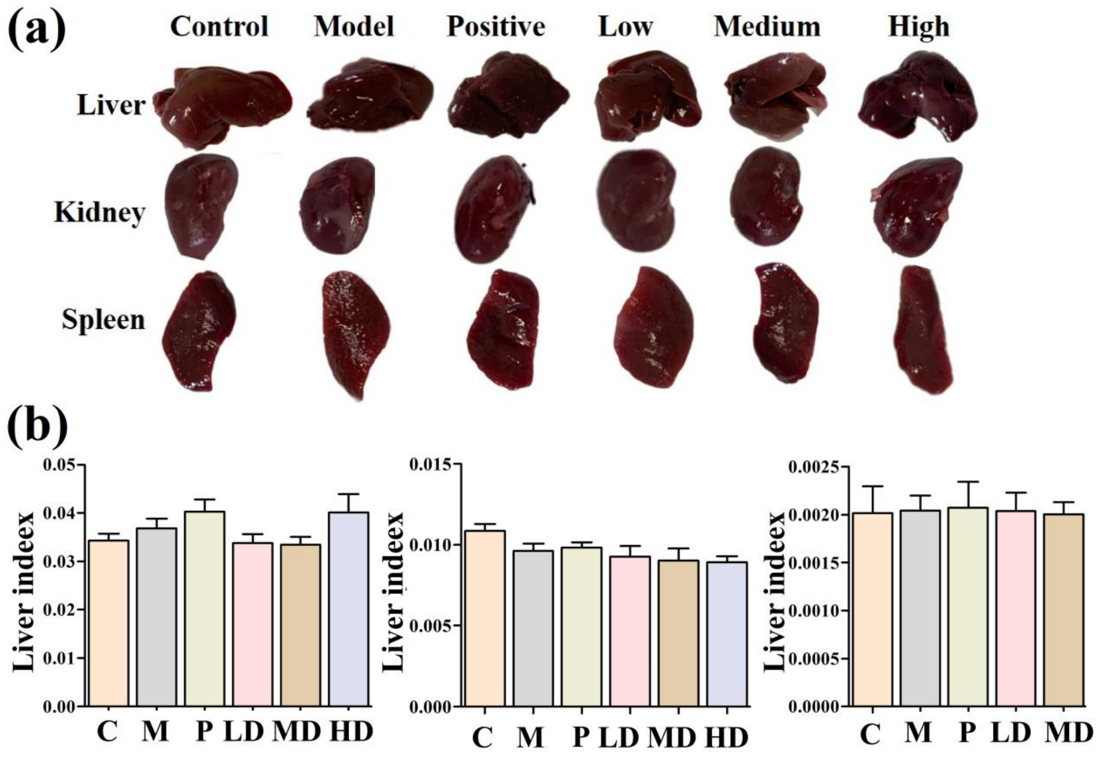

Figure S2 Toxicity evaluation of AA. (a) Images of liver, kidney and spleen of different groups of guinea pigs. (b) Liver index, kidney index and spleen index of different groups of guinea pigs. Model/M means model group, Positive/P means dexamethasone group, lOW/LD means low dose group, Medium/MD means medium dose group, and High/HD means high dose group. (n = 6 per group).

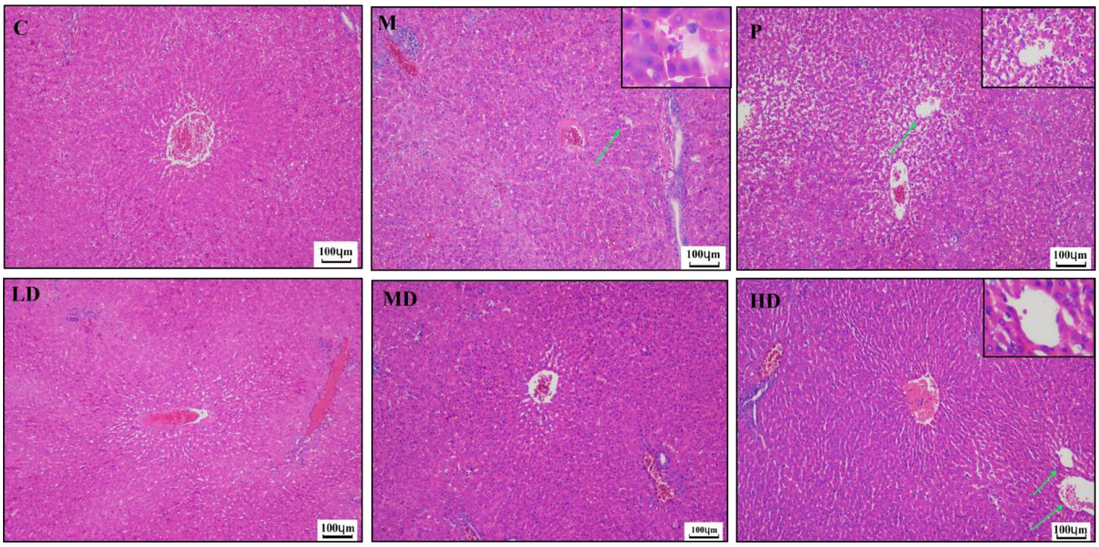

Figure S3 Toxicity evaluation of AA. HE staining of liver in different groups of guinea pigs. M mean model group, P means dexamethasone group, LD means low dose group, MD means medium dose group, and HD means high dose group.(n = 6 per group).

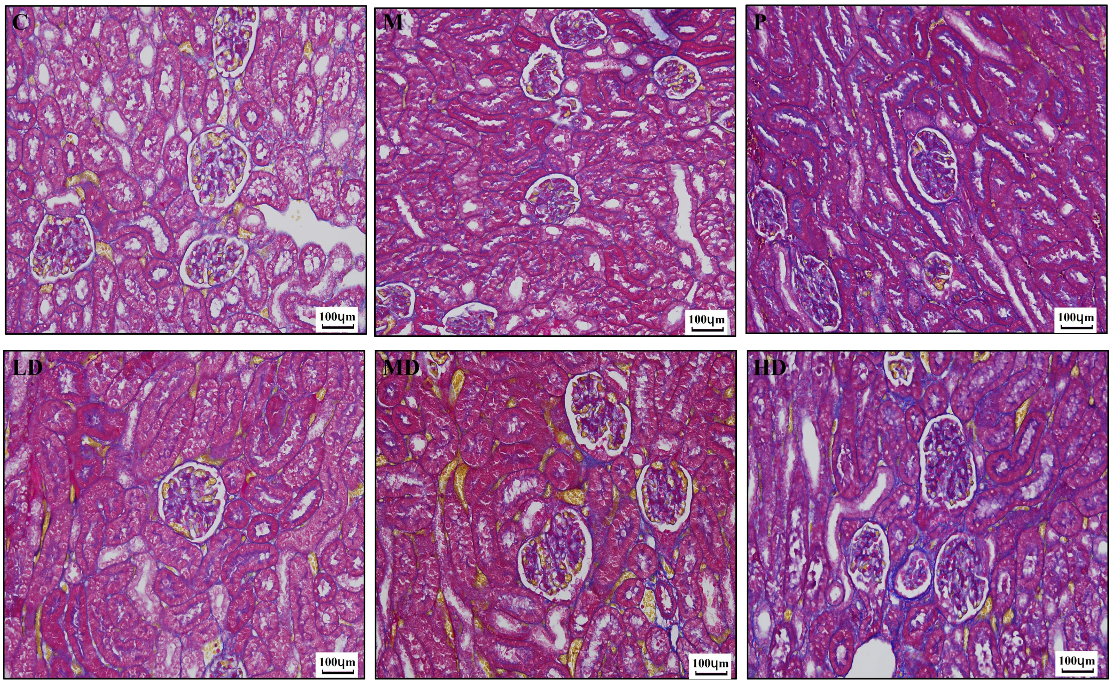

Figure S4 Toxicity evaluation of AA. Masson staining of kidney in different groups of guinea pigs.M mean model group, P means dexamethasone group, LD means low dose group, MD means medium dose group, and HD means high dose group. (n = 6 per group).
